# Progesterone-Related Immune Modulation of Pregnancy and Labor

**DOI:** 10.3389/fendo.2019.00198

**Published:** 2019-03-29

**Authors:** Nishel M. Shah, Pei F. Lai, Nesrina Imami, Mark R. Johnson

**Affiliations:** ^1^Department of Surgery and Cancer, Chelsea and Westminster Hospital, Imperial College London, London, United Kingdom; ^2^Department of Medicine, Chelsea and Westminster Hospital, Imperial College London, London, United Kingdom

**Keywords:** pregnancy, progesterone, PIBF, immune modulation, immune response

## Abstract

Pregnancy involves a complex interplay between maternal neuroendocrine and immunological systems in order to establish and sustain a growing fetus. It is thought that the uterus at pregnancy transitions from quiescent to laboring state in response to interactions between maternal and fetal systems at least partly *via* altered neuroendocrine signaling. Progesterone (P4) is a vital hormone in maternal reproductive tissues and immune cells during pregnancy. As such, P4 is widely used in clinical interventions to improve the chance of embryo implantation, as well as reduce the risk of miscarriage and premature labor. Here we review research to date that focus on the pathways through which P4 mediates its actions on both the maternal reproductive and immune system. We will dissect the role of P4 as a modulator of inflammation, both systemic and intrinsic to the uterus, during human pregnancy and labor.

## Introduction

Progesterone (P4) is a steroid hormone primarily produced by the ovaries, placenta, and adrenal glands in humans. It has an essential role in the establishment and maintenance of pregnancy as well as the onset of parturition (1–3). In the myometrium, P4 signaling has been attributed to the suppression of myometrial contractility by hindering pro-inflammatory cytokine production (4). P4 can also regulate local and systemic inflammation. In peripheral blood leukocytes, P4 appears to dampen pro-inflammatory cytokine production, which consequently reduces T-helper subtype differentiation and proliferation (5–8). Furthermore, P4 blocks natural killer [NK] cell degranulation and, therefore, cytolytic function (9).

The corpus luteum is a major source of endogenous P4 at the early stage of pregnancy prior to 12 weeks of gestation. Once pregnancy has established beyond 12 weeks of gestation, P4 production is sustained by the placenta at concentrations far greater than those in non-pregnant women (10). During the course of pregnancy, the production of P4 gradually rises to serum concentrations ranging between 175 and 811 nmol/L in the third trimester compared to non-pregnant levels of 1–2 nmol/L and 35–50 nmol/L in the follicular and mid-luteal phases of the menstrual cycle, respectively (10–12). P4 is likely to have both paracrine and endocrine roles during pregnancy, and its local effects in tissues in close proximity to the placenta are likely to utilize higher concentrations of this hormone.

The importance of P4 in the maintenance of pregnancy is highlighted by a systemic decline in its concentration prior to the onset of labor in most animal models (13, 14). Intriguingly, this is not evident in humans, and thus it is expected that a different mechanism of change in physiological P4 action is required to mediate human labor (15, 16). This has been supported by both animal and human studies that have used abortifacient regimens, either by (i) inducing a decline in systemic P4 levels, (ii) corpus luteum lysis or oophorectomy, or (iii) directly antagonizing the effects of P4 by the administration of an anti-progestin such as mifepristone (RU486) (17–22). It has been strongly suggested that the ability of RU486 to promote abortions and labor demonstrates such uterine processes are driven by “functional P4 withdrawal” in humans, which is mediated by altering the expression levels of P4 receptor (PR) isoforms and PR gene polymorphisms in reproductive tissues (23–25). In the immune system, indirect evidence of reduced P4 action at parturition is provided by studies that show a decrease in a lymphocyte-derived downstream immunomodulatory protein known as P4-induced-blocking factor (PIBF) (23, 26–28). Extracellular PIBF exerts an anti-abortive effect by interfering with availability of cytokines and NK cell activity (29); its content in urine has been observed to increase during pregnancy and dramatically decrease following childbirth. In pregnancy pathologies, including preterm labor (PTL) and pre-eclampsia, concentrations of maternal serum PIBF are low (30).

Animal pregnancy models have demonstrated that maintaining an elevated systemic concentration of P4 will prevent the onset of labor at term gestation and in models of inflammation-induced PTL (22). Evidence for P4-driven repression of human labor by pharmacological supplementation of this hormone is relatively less clear. Multiple studies have been carried out using different formulations and doses of P4 administered *via* different routes in women at risk of PTL, where some show benefit and others show no effect, but none show an adverse effect (31, 32). The most consistent beneficial response has been observed in women with a short cervix, who showed up to a 40% reduction in risk of PTL in a range of studies (33–35). The most consistent observation of an absence in response is in women with a multiple pregnancy, where P4 supplementation has demonstrated no effect in a number of studies (36–38). Failure of P4 supplementation to consistently prolong pregnancy supports the existence of functional progesterone withdrawal. At the same time, the findings of these studies suggest a process that links a shortening cervix to the onset of labor does seem to indicate responsiveness to P4 action.

Clinically, P4 is used to treat infertility, miscarriage and PTL, for which its efficacy has been extensively studied (39–41). The immunomodulatory effects of P4 in the endometrium and decidua are often suggested to promote embryo implantation and maintenance of pregnancy (40–42). P4 antagonism is an accepted approach to induce miscarriage or the onset of labor in situations of fetal malformation or loss. Typically, RU486 is administered before a prostaglandin (PG) analog to directly stimulate contractions. In the context of preventing miscarriage, the use of P4 is supported by the latest Cochrane review (42). In high risk groups for PTL, P4 is widely used to prevent premature onset of spontaneous labor and may significantly reduce an individual's risk of spontaneous preterm delivery, but the overall impact on absolute numbers of preterm births is small (0.01%); thus P4 does not greatly impact on perinatal mortality, low birthweight, or neonatal death (43). In this review we will introduce the effects of P4 on reproductive tissue and provide a detailed understanding of its role as a modulator of both inflammation and immune response.

## Receptor-Mediated Actions

### Nuclear PR

Nuclear PR (nPR) isoforms PRA and PRB represent the two major PR isoforms in reproductive tissues (44–46). Both PRA and PRB are transcription factors that directly bind to P4 (10, 47). Following P4-nPR interaction, these receptors can also modulate intra-cytoplasmic signaling cascades to indirectly affect transcription factor activity (48). In addition to regulation by ligand binding, the transcriptional activity of nPR isoforms can be controlled by their expression level, post-translational modification, and interaction with transcriptional co-regulators (10, 49).

Various co-regulators, both repressors and activators of gene transcription, can bind to both PRA and PRB to modulate their activity (50–53). PRA is a truncated from of PRB that has one less transcription activation domain, known as AF-3, at its N-terminus (46, 54). In an inactive state, both receptors reside in the cytoplasm while bound to chaperone proteins, which dissociates upon P4 binding to either (i) form dimers before translocating into the nucleus to interact with P4 response element (PRE) sequences at target gene promoter regions, or (ii) as a monomer that interacts with SRC-kinase complexes to activate extracellular signal-related kinases (ERK-1/2) and thus modulate transcription *via* the mitogen-activated protein kinase (MAPK) pathway (45, 55).

All reproductive tissues studied so far have been observed to express PRA and PRB in varying amounts, which is thought to result in differential expression of P4-responsive genes (56). It was previously thought that PRB has significantly greater transcriptional activity, which was attributed to its AF-3 domain located within the N-terminal 164 residues of its amino acid sequence (54). Whereas, PRA primarily functions as a ligand-dependent trans-dominant repressor of PRB activity (44). The ratio of PRA to PRB has consequently been considered to be an important factor for determining the status of P4 signaling. However, it is now known that PRA can also modulate transcriptional activity without the involvement of PRB, and they regulate the expression of distinct genes (4). In pregnancy, myometrial quiescence is suggested to, at least in part, be mediated by PRB and labor is associated with an increase in the PRA:PRB ratio that results in increased expression of pro-labor genes such as connexin-43 (Cx43), cyclooxygenase-2 (COX-2), oxytocin receptor (OTR) and nuclear factor κB subunit 2 (NF-κB2), which can potentiate myometrial contractility (4, 23, 57). PRA not only represses PRB activity but also, in its unliganded state, acts as a transcriptional activator of Cx43 (57–59). In addition to this, PRA is also capable of repressing the transcriptional activities of ER and GR, which illustrates the existence of cross-talk between their pathways (44, 60, 61).

A number of studies have debated the existence of nPR expression in peripheral blood lymphocytes in humans, where it seems they are either not expressed or only expressed at low levels (62–64). Studies of pregnant mice have reported nPR expression on T cells from its quantification at the RNA and protein levels, but nPR expression in humans remains a controversy (65). PBMCs from both non-pregnant and pregnant women have been shown to contain mPRs with the use of immunoperoxidase staining methods (66) and, more recently in T-lymphocytes from non-pregnant women, with the use of mRNA and radioactive P4 ligand binding measurements (62).

### Membrane-Associated PR

Non-classical PRs that are involved in extracellular signaling exist in addition to nPRs. The most commonly discussed types of non-classical PRs in pregnancy and parturition research are those that are integral membrane proteins, namely G-protein-coupled membrane progestin receptors (mPRs) and P4 receptor membrane components (PGRMCs). These have been classified to belong to the adipoQ receptor (PAQR) and b5-like heme/steroid-binding protein families, respectively (67). These two types of membrane-bound PRs differ from each other and to nPRs with regards to their tertiary structures, binding partners and intracellular signaling pathways.

The expression of mPRs was originally identified in fish species, where they were found to be particularly relevant in gamete function (68). They were subsequently identified in humans, but their localization in the plasma membrane has been debated and some studies suggest they reside in the endoplasmic reticulum instead (69–71). Five genetically distinct forms of mPRs are known, namely mPRα (PAQR7), mPRβ (PAQR8), mPRγ (PAQR5), mPRδ (PAQR6), and mPRε (PAQR9) (72). These receptors each have a seven-transmembrane domain structure that includes an extracellular P4-binding domain, which has high affinity but limited capacity for P4 binding (73). Activation of mPRs has been shown to both suppress adenylyl cyclase activity and enhance phosphorylation of myosin light chain protein in human myometrial cells (74). Karteris et al. also showed inhibitory G-protein signaling through mPR can regulate nPR transactivation to increase transcriptional activity of ligand-activated PRB (74). The authors propose that this effect diminishes when PRA:PRB ratio increases, suggesting that mPR may act synergistically with PRB (74). P4-mPR binding can also lead to activation of MAPKs (75), which can alter the phosphorylation status of transcription factors. Kinases of the MAPK pathway have been suggested to play roles in T lymphocyte activation (76).

PGRMCs are encoded by two genes, PGRMC1 and PGRMC2, and their expression was originally discovered in rat and porcine hepatocytes (77, 78). Since then, PGRMC1 has been isolated from a range of human cells and tissues, including neuronal, breast, and reproductive tissues (62, 79–81), and have been found to localize to both the plasma membrane and organelle membranes (82–85). The ability of PGRMC1 to bind to P4 has been demonstrated with the use of radioactive P4 ligand binding measurements from porcine liver membrane fractions and, more recently, spectroscopy (77, 86). Both PGRMC1 and PGRMC2 are single membrane-spanning receptors that bind to heme and can interact with SERPINE1 mRNA-binding protein (SERBP1) to enable activation of second messenger pathways (86, 87). In granulosa cells, for example, PGRMC1 forms a complex with SERBP1 on the plasma membrane, and P4 binding to PGRMC1 in these cells can potentiate P4-associated anti-apoptotic effects (88). PGRMCs can also activate PKG activity upon P4 binding, which is also not a feature of classical mPRs (86, 89). In the endometrium, expression of PGRMC1 is upregulated in the proliferative phase of the menstrual cycle, as well as the maternal-fetal interface and embryonic/fetal trophectoderm in pregnancy (87, 90), which suggests that PGRMCs are likely to have a role in the cell cycle within these tissues of the reproductive system.

**Figure 1:**
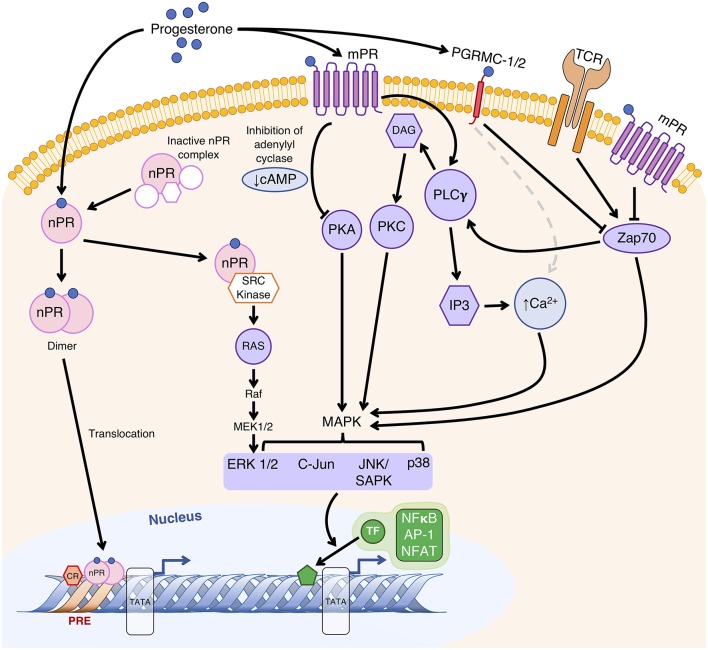
Proposed mechanisms of P4-regulated gene transcription to modulate T cell function. Classically, extranuclear nuclear P4 receptors (nPR) exist in an inactive state until P4 binding, after which it forms a dimer and translocates to the nucleus to bind to P4 response element (PRE) sequences within gene promoter regions to alter their transcriptional activity. Alternatively, as a monomer, nPR-P4 acts *via* the Src kinase to activate the MAPK cascade. P4 bound to membrane P4 receptors (mPR) alters gene transcription regulated by second messengers (cAMP and Ca^2+^) and their associated extranuclear kinases (PKA and PKC) *via* the MAPK signal transduction cascade to result in phosphorylation of nuclear transcription factors (TF). Membrane-bound P4 receptors mPRs and P4 receptor membrane components (PGRMC) likely affect T cell receptor (TCR) signal transduction by modulating the activities of MAPKs through Zap70, as well as Ca^2+^ mobilization caused by phospholipase Cγ (PLCγ)-driven production of diacylglycerol (DAG) and 1,4,5-trisphosphate (IP3), which lead to modulation of pro-inflammatory gene expression and T cell activation *via* transcription factors NF-κB, AP-1 and NFAT. Adapted with permission from Mesiano et al. (55), and Mani et al. (94).

Pregnant women have been shown to express mPRs and PGRMCs in T cells during pregnancy (7, 91, 92). P4-driven modulation of T cell receptor (TCR) signal transduction and T cell function (7, 62–64) are potentially mediated by membrane-bound PRs in human T cells. The downstream pathways following TCR engagement involves MAPK phosphorylation and inositol trisphosphate (IP3) production, the latter of which promotes the release of Ca^2+^ stored in the endoplasmic reticulum (93). On T cells, however, P4 binding to PGRMC1 and classical mPRs results in increased intracellular Ca^2+^ mobilization and reduced phosphorylation of ζ-chain-associated protein kinase 70 (Zap70) to modulate T cell activation and TCR-mediated immune responses (Figure 1) (63). Both pathways modulate phosphorylation of transcription factors that include NF-κB, activator protein 1 (AP-1) and nuclear factor of activated T cells (NFAT), which are associated with pro-inflammatory gene expression as well as T cell activation and proliferation (Figure 1) (93).

In addition to mPRs and PGRMCs, it has also been suggested that OTR is a target for progestogen modulation. In human myometrium, 5β-dihydro-progesterone, but not P4, can inhibit oxytocin binding *in vitro* (95). OTR receptors are also expressed on T cells, where their activation has been shown to stimulate Ca^2+^ release in lymphocytes and, more importantly, P4 inhibited estradiol (E2)-induced expression of OTR mRNA in T cells (96).

### Nuclear GR

Although P4 signaling is primarily nPR-mediated, P4 has also been shown to bind weakly to the nuclear glucocorticoid receptor (nGR), and this interaction may be an important mechanism of anti-inflammatory action by P4 in reproductive tissues (97). In primary myometrial cell cultures, we have previously shown that P4 acts *via* nGR, rather than PR, to suppress NF-κB dependent inflammation (98, 99). Furthermore, glucocorticoids have significant anti-inflammatory effects in immune cells, such as inhibiting the transcription of pro-inflammatory cytokines and chemokines in macrophages and increasing regulatory T cell (Treg) proportions in pregnant mice (100, 101). Promiscuous binding of P4 to nGR is likely to drive some of the previously observed glucocorticoid-like effects in reproductive tissues and immune cells. Recently, *in vitro* treatment of spleen-derived T cells obtained from nGR and nPR conditional knock-out mice during pregnancy demonstrated P4-induced T-cell death is mediated *via* nGR and not nPR (102); the authors of this study recommended *in vivo* validation to assess the impact of hormone bioavailability on P4-nGR binding on T-cell survival. Glucocorticoid excess in early pregnancy is associated with adverse effects on placental function, and it has the potential to convert myometrium from a quiescent state to an estrogen-primed contractile state in late pregnancy, by upregulating COX-2 expression in the amnion and placenta to induce parturition (103–106).

Like other members of the steroid hormone receptor family, nGRs are ligand-dependent transcription factors, which were first identified in thymus cells but have subsequently been observed in other human cell types (107). In a similar fashion to nPR, unliganded nGRs reside in the cytoplasm bound to heat shock proteins (HSPs) to form an inactive complex. The interaction between nGR and HSPs is disrupted by glucocorticoid binding to nGR, which then allows nGR to interact with its DNA-binding sites within the nucleus (107). The nGR family comprises a number of isoforms, but there are two principle variants, GRα and GRβ, which share a similar relationship to that between PRA and PRB because GRβ is thought to be a negative regulator of GRα (108, 109). Much in the same way as PR, the nGR isoforms have varied tissue distribution and are responsible for regulating the expression of distinct genes (110). For the most part, these regulatory processes constitute the genomic actions of glucocorticoid-GR engagement. However, other signaling cascades independent of nGR contribute to glucocorticoid activity, including interaction with membrane-localized GR, and these represent alternative mechanisms of action (111, 112).

### Membrane-Associated GR

Similar to membrane-bound PR receptors and their interaction with P4, rapid effects of glucocorticoids are thought to occur *via* membrane GR (mGR). These receptors were first reported in a mouse lymphoma cell line and subsequently, using amphibian neuronal tissue, found to be coupled to G proteins (113–115). Both nGR and mGR appear to share components of the NR3C1 gene (116). At the same time mGR expression can be prevented *in vitro* using brefeldin A, a Golgi apparatus transport inhibitor, which indicates a translocation of mGR to the plasma membrane (117, 118). Treatment of a human embryonic kidney cell line (HEK 293T) *in vitro* with glucocorticoids results in activation of the p38 MAPK signaling and pro-apoptotic activities (117, 119). In mice, dexamethasone binding to membrane fractions prepared from lung tissues has been assessed to demonstrate, in part, the existence of mGRs (120), but their ability to bind P4 has yet to be shown. To date, mGR expression has been demonstrated in human monocytes and B cells but not T cells (118, 121). Proteomics-based pathway analysis of a human acute lymphoblastic leukemia T-cell line (CCRF-CEM) stimulated with membrane-impermeable cortisol has identified RhoA signaling to most likely be activated upon mGR activation (122).

## P4 Suppression of Pregnancy-associated Inflammation in Reproductive Tissue

In preparation for pregnancy, the maternal immune environment adapts to tolerate the semi-allogenic fetus and these adaptations are thought to be, in part, anti-inflammatory. The overall impact is likely to be mediated by the endocrine system *via* changes in concentrations of P4, E2 and human chorionic gonadotropin (hCG) as well as influenced by previous maternal exposure to paternal factors (123, 124). In fact, preparation for pregnancy begins during the menstrual cycle, where E2 and P4 optimize the uterine environment and vaginal mucosa for conception and implantation (123, 125). However, as a consequence, this optimum time to establish pregnancy is also the most vulnerable period for would-be mothers because the anti-inflammatory effects of P4 modulate the innate and adaptive immune systems in a way that results in limited responses to sexually transmitted pathogens (125–127). Locally, an increase in P4 activity during the luteal phase also directs endometrial decidualization, which is a key to enable successful trophoblast invasion and placentation in early pregnancy. P4 also stimulates other local factors in the reproductive tract, such as glycodelin A and transforming growth factor β (TGF-β); these have tolerogenic biological activities, particularly in early pregnancy (128, 129).

Glycodelin-A is a secretory glycoprotein that is synthesized in endometrial glands and its secretion is related to P4 activity. Animal *in vivo* studies have demonstrated that the production of a glycodelin homolog can be increased following P4 stimulation and, following pregnancy, by hCG (130). It has a range of immunomodulatory properties that enhance maternal tolerance, which include reduced NK cytotoxicity, preferential apoptosis of Th1 subsets, and induction of tolerant dendritic cell (DC) phenotypes and Th2 cytokine production (128, 131–133).

In mammalian tissues, the TGF-β superfamily of cytokines includes multiple sub-groups of polypeptide ligands. The TGF-β homologs form one of these sub-groups, which consists of TGF-β1, TGF-β2, and TGF-β3 (134, 135). Murine genetic knock-outs for each of these TGF-β homologs have demonstrated their differences in physiological roles, along with demonstration of their differential expression patterns during embryogenesis, and chimeric mice for TGF-β1 knock-out and TGF-β2 knock-in show their functions are not fully interchangeable (136, 137). In decidua, TGF-β appears to regulate trophoblast invasion and placentation, and to be expressed in syncytio, chorionic, and extra-villous trophoblast (138, 139). During trophoblast invasion, TGF-β has anti-proliferative, anti-invasive and pro-apoptotic actions, that regulate tissue growth and invasion, tissue remodeling, and angiogenesis required for successful placentation (140). In mice, anti-CD3 stimulation in the presence of TGF-β mediates immune suppression *via* the induction of forkhead box P3 (FoxP3) expression in naïve T cells; FoxP3 is a specific lineage marker with functional relevance for Tregs, since mutations of the FoxP3 gene result in loss of Treg function, and different isoforms of FoxP3 confer different suppressive ability (141–144). Furthermore, TGF-β polarizes DC maturation toward a tolerogenic phenotype (145).

Following implantation, concentrations of P4 increase, halting pro-inflammatory and cytotoxic leukocytes recruitment *via* GR suppression of cytolytic function mediated by PIBF blockade of degranulation (123). Once pregnancy is established, the anti-inflammatory effects of P4 observed prior to implantation are enhanced. In human and animal models, P4 has been shown to have both systemic and tissue-specific anti-inflammatory effects, repressing both NF-κB and MAPK pathways to downregulate COX-2 expression (4, 98, 146–148). This represses local PG production that would otherwise increase placental corticotropin release hormone (CRH) to potentiate NF-κB signaling to drive pro-inflammatory gene expression and leukocyte recruitment (149). Additionally, P4 has been shown to halt IκBα degradation, which prevents NF-κB activation and so downregulate the expression of labor-associated genes such as OTR and Cx43, and inhibit pro-inflammatory cytokine production in reproductive tissues and immune cells (150–153). However, P4 is far less effective once inflammation is established, which may be attributed to the expression ratio of PR isoforms in reproductive tissues (154, 155).

Primary myometrial cell cultures have previously been used to show that altering PR expression to favor PRA leads to increased P4-driven pro-inflammatory gene expression, whereas the reverse is true if PRB expression is dominant (3, 4). Small alterations in placental PR levels can modulate Th1 cytokine production and subsequently PTL risk (156). As discussed above, differential expression of PR isoforms is thought to contribute to the onset of labor (57). Murine experiments have shown that, during both pregnancy and labor, PRB and NF-κB pathways remain in opposition, and responsiveness to exogenous P4 may attenuate maternal inflammatory processes thereby tipping the balance to favor PRB (157, 158). Outside the maternal–fetal interface, P4 concentration in the systemic circulation tends to be relatively low in humans and its immunomodulatory effects may be determined by lymphocyte sensitivity to the hormone (159). Our own analysis of P4 effects during pregnancy suggest that the systemic effects of P4 become less pronounced from ~34 weeks of gestation (160), which coincides with increased expression of activation markers on circulating and decidual T cells in late pregnancy and prior to the onset of labor (161, 162).

Cervical ripening occurs in the presence of increased cervical vascular permeability, inflammatory cytokine and PG production, macrophage numbers and a reorganization of collagen cross-linking to prepare the cervix to open (163). P4 is thought to prevent some of these changes. However, RU486 initiates uterine activity but does not promote cervical ripening in rhesus macaques (164), and RU486 can act as a PR agonist in the event of, for example, changes to protein expression ratio of PR coactivators to corepressors or the absence of P4 binding (53, 165–168). On the other hand, P4 treatment of endometrial cell cultures has been shown to upregulate collagen accessory proteins that, if directly translatable to the *in vivo* condition, would contribute to the maintenance of cervical structure (169, 170). The effect of P4 on the cervix may be mediated through reduced inflammatory cell infiltration (171, 172). For example, analysis of murine cervical leukocyte infiltration has previously shown a dominance of monocytes after lipopolysaccharide (LPS) exposure and neutrophils after RU486 exposure, which suggested that P4 may influence neutrophil chemotaxis (173).

PTL is often associated with a reduction in resident inflammatory cells, such as macrophages, when infection is a factor of consideration (174). Our own unpublished observations from longitudinal analysis of cervical mucus obtained from women with a high risk of preterm delivery or short cervix, whilst receiving vaginally-administered P4 treatment, found an increase in populations of total innate cells, which consisted of predominantly neutrophils as well as stable proportions of monocytes and macrophages. This suggests P4 may act to maintain the leukocyte composition in cervical mucus to reduce susceptibility to infection-mediated PTL. However, the protection of cervical mucus during pregnancy may not be entirely reliant on its immunological properties; its permeability to microbial infiltration or similar aspects may have a role to play instead (175).

## P4 Modulation on Innate Immune Function

The reports mentioned above suggest that P4 reduces inflammatory cell infiltration into the cervix and cervical mucus. Furthermore, *in vitro* studies have demonstrated P4 inhibition of human neutrophil degranulation and free radicals generation, whilst also promoting an increase of tolerant CD4 T cells expressing glycoprotein A repetitions predominant (GARP) protein and with a CD127^lo^FoxP3^+^ phenotype that can produce interleukin (IL-) IL-10 and IL-17 as well as vascular endothelial growth factor (VEGF) (176, 177). P4 can inhibit mature DCs and DC-mediated proliferation of T cells, favoring immature DCs that promote immune tolerance (10). The addition of P4 to *in vitro* co-culture of rat DCs and T cells can reduce T cell proliferation in response to LPS (178). Tolerant DCs have reduced expression of co-stimulatory markers CD40, CD80, and CD86, which are needed for T cell activation and secrete the immune regulatory cytokine IL-10 that potentiates Treg suppressive activity (10, 179, 180).

As well as promoting tolerant immune cells, progestogens also suppress the activity of potent type I interferon (IFN)-producing DCs. For example, by binding to GR, medroxyprogesterone acetate (MPA), but not norethisterone (NET) or levonorgestrel (LNG), can reduce human IFN-α production by plasmacytoid DCs (pDCs) in response to inactivated virus or TLR ligands *in vitro* in a concentration-dependent manner (181). IFN-α can potentiate the maturation of conventional DCs (cDCs) that interact with T cells, and are able to induce IL-10-producing Tregs (182).

*In vitro* experiments using PIBF-expressing human leukemia cell lines have demonstrated that P4 may actually enhance the innate immune response by increasing expression of TLRs, in particular TLR4, along with other proteins involved in their signaling (183). Observations by Schatz et al. showed in human decidua that TLR4 expression is significantly increased in first trimester DCs when compared with luteal phase or pre-decidual stromal cells. Furthermore, the expression of TLR4 on these DCs was preferentially increased in the first and third trimesters of pregnancy, but remained greater than the expression on interstitial trophoblasts irrespective of gestation (184). Similarly, Ziegler et al. showed, using PBMCs, that IFN-α production by TLR7-stimulated pDCs was low in early pregnancy relative to non-pregnant controls and subsequently increased with gestation, whereas TLR4-induced TNF-α production by monocytes was greatest in early pregnancy and subsequently decreased (185). These results indicated that although DCs in pregnancy have an immune tolerant phenotype with a reduced ability to activate T cells, their increasing TLR expression suggests they can still function effectively against invading pathogens to initiate an immune response.

Although TLR4 primarily interacts with LPS, it can also interact with host-derived molecules released during tissue damage in, for example, non-infective PTL and pre-eclampsia (184). It seems DC modulation by P4 may be designed to enhance their ability to respond to innate immune stimuli whilst suppressing T cell activation in order to promote fetal antigen tolerance. This has been demonstrated in pregnant mice, where P4 treatment can increase myometrial IFN-γ-expressing neutrophils as well as cervical active matrix metallopeptidase 9 (MMP9)-positive neutrophils and monocytes (158). The secretory endometrium in non-pregnant women is also associated with increased TLR gene expression, which is likely to reflect a specific innate protective consequence of endocrine immunomodulation (186).

Injectable P4-based contraceptives in non-pregnant women are associated with a fall in chemokines monocyte chemoattractant protein 1 (MCP-1), macrophage-derived chemokine (MDC) and fractalkine, along with cytokines IL-15 and IL-12p40, and the platelet-derived growth factor (PDGF)-AA in cervico-vaginal fluid (187). Both MCP-1 and MDC are chemokines primarily involved in the recruitment of monocytes, monocyte-derived DCs and NK cells to sites of inflammation, although they are also thought to attract memory T cells and specifically Th2 subsets in the presence of antigen-induced inflammation (188–190). Fractalkine can attract and activate NK cells when present at sites of inflammation and may enhance the recruitment of cytotoxic T cells in the endometrium (191, 192). Similarly, IL-12p40 attracts macrophages and promotes the migration of activated DC, whereas IL-15 facilitates B-cell activity, NK cell generation and maintenance, and supports long-lasting CD8 T cells (193, 194). Platelet-derived growth factor (PDGF)-AA is a pro-angiogenic factor produced by various cell types, such as platelets, macrophages, endothelial cells and smooth muscle cells, and it has been shown to have a role in cellular proliferation, angiogenesis, inflammation, and tissue repair (195). In the upper genital tract, P4 also has anti-inflammatory effects, where P4 and E2 together, but not P4 alone, decrease TLR expression in cultured human fallopian tube epithelial cell lines (196). Similar effects have been observed in the amniotic membrane epithelium, where *in vitro* P4 treatment of explants suppressed LPS-induced release of IL-6, IL-8, TNF-α, and IL-10 (197) in tissues obtained from non-laboring pregnant women.

In addition to tissue-specific effects, P4 has a range of immunosuppressive effects on other innate leukocytes. P4 can suppress macrophage activation induced by both LPS and IL-4 *in vitro* and reduce both nitric oxide synthase 2 (iNOS) and arginase activity in a dose-dependent manner irrespective of the stimuli used (198). Furthermore, P4 pre-treatment of murine macrophages can inhibit LPS and CpG oligodeoxynucleotide (ODN)-induced IL-6 and NO production, as well as upregulate TLR4 expression (199), but it is uncertain whether P4 is acting by nPR. Jones et al. (200) postulated that these effects on murine macrophages are mediated *via* the GR receptor and, more recently, Lu et al. (201) suggested P4 may be acting *via* mPR; although both studies did not use pregnancy models and the macrophages were obtained from male mice. P4 may modulate the differentiation of macrophages into tolerant phenotypes in the uterus during early pregnancy, where immune mediators such as HLA-G, and cytokines IL-4, IL-10, and M-CSF are thought to regulate macrophage differentiation to promote phenotypes tolerant to the invading trophoblast tissue (202–206). Typically, macrophages differentiate into M1 or M2 phenotypes depending on a classical or alternative activation pathway, both of which can be promoted by microbes and microbial products, but each has a specific cytokine stimulation repertoire that is either pro-inflammatory (IFN, TNF) or anti-inflammatory (IL-4, IL-13) (207, 208); M1-M2 dichotomy is thus considered to be analogous to T-helper (Th1-Th2) phenotypes. MPA is a potent progestin that also has glucocorticoid activity. Tsai and colleagues have demonstrated that *in vitro* MPA treatment of a human monocyte cell line can stimulate differentiation to produce immune-tolerant M2 macrophages, which are able to induce decidualization of endometrial stromal cells, potentiate trophoblast invasion and resist responses to TLR agonists, thereby creating an environment typically associated with successful pregnancy (209).

## P4-Modulation of Adaptive Immune Responses and Overlapping Systems

Human PBMCs are thought to express a number of steroid receptors, such as PR, GR, and E2 receptor (ER) (210, 211). However, the majority of P4 effects on immune function are mediated *via* GR and PR. For example, CD8 T cells express nPRs, which can be increased by immunotherapy for successful treatment of recurrent miscarriages (66, 212, 213). Whereas, mPRs have been detected in Tregs isolated from pregnant women, which has been observed to increase in expression with advancing gestation and subsequently decrease at labor (214). Steroid hormone sensitivity of Tregs has been demonstrated by a study on patients with multiple sclerosis (MS), whereby *in vitro* treatment with E2, P4 and the combination of both hormones in mixed lymphocyte reaction (MLR) cultures enhanced Treg suppressive activity for both MS and healthy control subjects to a similar extent (215). The findings of this study suggest that Tregs may not increase in number *in vitro*, but their suppressive activity may be increased in response to proliferating responder T cells stimulated by allo-antigen in the presence of E2 and P4. In mice, GR engagement increases Treg immunosuppressive function during pregnancy, and P4 binding to nGR may mediate such an effect in humans (101, 102). GR agonists are also potent direct suppressors of Th1 differentiation in mice (8), and inducers of T cell apoptosis (216).

During mouse pregnancy, systemic and uterine Treg proportions and their suppressive activity have been shown to be enhanced with P4 supplementation and blocked by RU486, but this does not prevent spontaneous fetal loss in abortion-prone mice (65, 217). Findings from the latter study relate to early and mid-pregnancy, when the maternal response to the initial surge of fetal antigen is vital for maintaining pregnancy, and the murine abortion model used was previously found to produce poorly functioning maternal Tregs that contributed to their high susceptibility to miscarriage (218). Thus it is possible that expansion of these Tregs by P4 would not be sufficient to improve tolerance to fetal antigen. This is supported by the observation that adoptive transfer of Tregs from normal pregnant to abortion-prone mice in early pregnancy improves placentation and reduces reabsorption rates (218–220). Vaginal P4 supplementation in mice can increase decidual Tregs and pre-treatment with P4 is associated with protection against endotoxin-induced PTL (158).

In humans, however, our own *in vivo* study showed that Treg proportions gradually fall during the course of pregnancy in women who were given P4 treatment, but their cell-mediated IL-10 responses were comparable to untreated pregnancies (160). Mjösberg et al. showed that P4 reduces functionally suppressive Tregs in second trimester human pregnancies (221), whereas our findings showed an initial peak in Treg proportions compared to non-pregnant controls, followed by a steady decline in late pregnancy (160). We proposed that this fall may reflect the overall anti-inflammatory effect of P4 and suggested that alternative immunomodulatory pathways other than that provided by Tregs are at play. However, Tsuda et al. have shown that effector Tregs in the decidua are clonally expanded in the third trimester of pregnancy (222). Therefore, alternative explanations for our own observations from peripheral blood could be the selective recruitment of fetal-specific Tregs to the maternal-fetal interface or relative expansion of other leukocyte populations.

In human T cell clones, P4 modulates resting peripheral blood T cell differentiation into Th1, Th0 or Th2 clones, promoting Th2 subsets as shown by the increased production of IL-4 (10). P4 also induces enhanced production of IL-5 and IL-4 by T cells, as well as detectable amounts of IL-4 in Th1 cell lines, enabling Th2 dominance in pregnancy (10). Increased *in vivo* (gestation-related) and *in vitro* extracellular P4 concentrations can result in the suppression of IFN-γ production in human CD8 T cells during pregnancy, which has been attributed to hypermethylation of the IFN-γ gene promoter based on findings from pregnant mice (7, 223). In murine pregnancy, galectin-9 (Gal-9) is a biologically active protein found in the cytoplasm and extracellular environment that also has a unique binding specificity for glycans (224). Gal-9 can promote the induction of Tregs and apoptosis of Th1 cells, by binding to T-cell immunoglobulin and mucin-domain containing-3 (TIM-3) expressed on the surface of terminally differentiated Th1, but not Th2, cells and its binding downregulates Th1 and Th17 responses (225, 226). The gal-9/TIM-3 pathway has been shown to be susceptible to P4 immunomodulation in mice placenta. It has been proposed that P4 alters gal-9 and TIM-3 expression by NK and T cell subsets, along with CD107a expression by TIM-3^+^ dNK cells and dNKT cells (227). CD107a is a useful surrogate marker for NK cell activity because it correlates well with both cytokine secretion and target cell lysis (228). Arruvito et al. have shown that peripheral blood NK cells express classic nPR, and this expression is restricted to mature KIR^+^ phenotypes (229). P4 activity in these cells is associated with an increased susceptibility to both suppression of IL-12-induced IFN-γ secretion and caspase-dependent apoptosis (229). In contrast, human uNK cells have no PR expression and P4 does not have direct effects on their biological activity (230, 231). Murine work suggests that these effects are predominantly mediated by PIBF (232–234).

The menstrual cycle provides a human model for the *in vivo* effects of P4 that is alternative to that of pregnancy. Despite slightly conflicting data across studies, they have shown overall that the P4-rich luteal phase of the menstrual cycle is associated with a decline in Tregs, leukocyte proliferation, and IFN-γ production, as well as a shift toward Th2 cytokine production (235–240). There is evidence to suggest the effect of Tregs in this context and during pregnancy may be influenced by previous exposure to paternal antigens, and therefore greater parity and sexual activity with a male partner prior to the index pregnancy may act to enhance P4 action (236, 241, 242). P4 may also suppress primary immune responses by naïve T cells, since functionally active nPR receptors are expressed in the thymus and these are thought to be involved in thymic involution during pregnancy (1, 243).

P4 and low dose E2 administration to female mice have been associated with reduced B cell lymphopoiesis, which suggests that B-cell development in bone marrow is subject to P4 immunomodulation (244). Murine B cells have been shown to express nPR at the mRNA level and the preference for Th2-skewed responses with P4 interactions suggest that P4 is likely to influence the humoral immune response. B cell and endometrial cell *in vitro* co-culture in the presence of P4 has been shown to suppress B cell antigen presentation due to reduced expression of co-stimulatory molecules CD80 and CD86 (245). However, activation-induced deaminase mRNA synthesis, which is required to promote a diverse immunoglobulin repertoire and achieved by somatic hypermutation and class switch recombination among other mechanisms, in activated mouse splenic B cells is reduced with P4 treatment (246, 247). In addition, during respiratory influenza A infection, female mice treated with P4 and levonorgestrel produce fewer antibodies in sera and locally in bronchial-alveolar lavage fluid (248). Therefore, it is surprising that whilst P4 promotes a Th2-dominant immune profile, it also negatively regulates the production of high-affinity antibodies. With significant fetal antigen exposure in pregnancy, however, this is probably a protective feature. The Th2 bias that is thought to be characteristic of P4 treatment is in fact associated with increased asymmetric antibody production, which may have a protective role during pregnancy (249).

Despite the wealth of data available, the exact mechanism of P4 action on lymphocytes is still poorly understood and this can be partly attributed to varied outcomes of studies that examined their expression of nPRs (250). The observed rapid actions of some steroid hormones fail to agree with a classical genomic mechanism of action through nPR, which is commonly associated with a longer lag time (250, 251), and thus mPRs are more likely to be the mediators of P4 activity in these cases (62). Of note, a study by Dosiou et al. on lymphocytes taken from non-pregnant women of reproductive age demonstrated mPRα expression on CD8^+^, but not CD4^+^, cells, which can be increased by a P4-dominant environment like that of the mid-luteal phase of the menstrual cycle (62); the same was not true for mPRβ expression. Their results also demonstrated specific binding of P4 to plasma membranes of T cells and showed a concentration-dependent activation of inhibitory G-proteins in Jurkat cells (62). Chein et al. showed that RU486 suppressed P4-mediated rapid increases in Ca^2+^ in human T cells expressing mPRs, but enhanced the inhibitory effect of P4 on phytohemagglutinin (PHA)-induced cell proliferation (252). Therefore, it seems likely that the mechanism by which P4 elicits its activity may vary with different lymphocyte subsets and include both genomic and non-genomic pathways *via* different receptors and downstream effects.

## P4-Induced Blocking Factor (PIBF)

PIBF is a PR-regulated gene and potent immune-modulator (253). It is expressed most abundantly as a protein composed of 757 amino acid residues and has a predicted molecular mass 90 kDa (254). This form has been shown to be associated with the centrosome and may have a role regulating the cell cycle (255). However, several other shorter splice variants have been recognized with tissue-specific intra- and extra-cellular expression (255–257). These short isoforms are also thought to be ligands for the PIBF receptor (256, 258). Highly proliferative cells such as trophoblast, mesenchymal stem cells, and tumor cells all express PIBF (257, 259, 260). PIBF expression is hormone-dependent, and the expression of PIBF is increased during pregnancy (159). Studies using murine models of hematological cancers have shown that PIBF mRNA levels are increased and decreased in the presence of P4 and RU486, respectively (183, 212). The PIBF receptor appears to be glycosylphosphatidylinositol (GPI)-anchored and can form a heterodimer with the IL-4 receptor (IL-4R) *via* the latter's α-chain component (261). PIBF is thought to mediate a large proportion of P4-regulated effects in lymphocytes during pregnancy (6, 262), which include cytokine synthesis and subsequent T-helper subtype differentiation and proliferation (5, 6). T cell effector functions modulated by PIBF include Th2-type cytokine synthesis, suppression of cytotoxic T and NK cell activity, and arachidonic acid synthesis (5, 9, 27).

### P4-Dependent Regulation of PIBF Expression

PR^+^ T cells in the peripheral circulation in pregnancy are predominantly γδ^+^ or CD8^+^ T cells that express PRs once activated after immune recognition of fetal and placental-derived antigens, which occurs in a hormone-independent manner (66, 263, 264). The quantity of γδ^+^ PR-expressing T cells in peripheral blood in notably higher in pregnant than non-pregnant women, though comparatively less than decidua where they comprise a large proportion of all T cells during pregnancy (264). Importantly, γδ T cells interact with unprocessed foreign antigens in an MHC non-restricted manner and with non-polymorphic Class I or Class II like molecules (265, 266). Note that PR expression can be stimulated by any allo-antigen, not necessarily feto-placental, and T cells can exhibit increased PR expression following organ transplantation and blood transfusion in non-pregnant women (267). Maternal interaction with fetal antigens during pregnancy is a potentially important step in PR expression and murine studies have suggested that placenta-derived non-classical HLA molecules, such as HLA-C, serve to present fetal/paternal antigens to maternal lymphocytes (268). In pregnancy, the trophoblast, which forms the interface between maternal and fetal compartments, expresses a number of non-classical MHC class I molecules, including HLA-C, HLA-E, and HLA-G proteins. The expression of HLA-G, in particular, is enhanced in the presence of P4 and cytokines IL-10 and IFN-γ (269, 270).

Our own studies have shown that PIBF expression on CD8^+^TCR-γδ^+^ T cells is increased during healthy pregnancy, independent of *in vitro* P4 stimulation, and this appeared to be dominant in the second trimester of pregnancy (271). It is nevertheless likely that our observation of a gestational variance represented relative differences in endogenous P4 and fetal antigen exposure prior to culture. On the other hand, Cohen et al. proposed that P4 supplementation and endogenous elevation of P4 during the menstrual cycle were sufficient to induce a rise in PIBF concentrations, but the effects of paternal antigen exposure were not discussed (254, 272).

### Suppression of Pro-inflammatory Cytokines

Szekeres-Bartho et al. were the first to demonstrate that P4-treated lymphocytes obtained from healthy pregnant women released PIBF, which was able to block both cytotoxic activity and prostaglandin F2α (PGF2α) synthesis in lymphocytes; women with clinical symptoms of threatened preterm delivery failed to synthesize PIBF (27). In this instance, PIBF functions like a cytokine and binds to its GPI-anchored protein receptor at the plasma membrane to mediate intracellular effects. Since then, other studies have demonstrated that PIBF concentrations in the urine and plasma of women who experienced spontaneous preterm birth is significantly reduced, and that cytokine production is skewed toward a pro-inflammatory profile with reduced IL-10 and increased IL-6 and IFN-γ (5, 273). Additionally, murine experiments that tested the influence of embryo-derived extracellular vesicles showed those that contained PIBF increased IL-10 production by CD8 T cells *in vitro* (274). Thus, PIBF appears to be important for maintaining pregnancy by contributing to the mechanisms of immune tolerance. However, not all lymphocytes express either PIBF or PR (229, 262, 275–277), and PIBF may exert some if its actions *via* mechanisms independent of PR (274).

**Figure 2:**
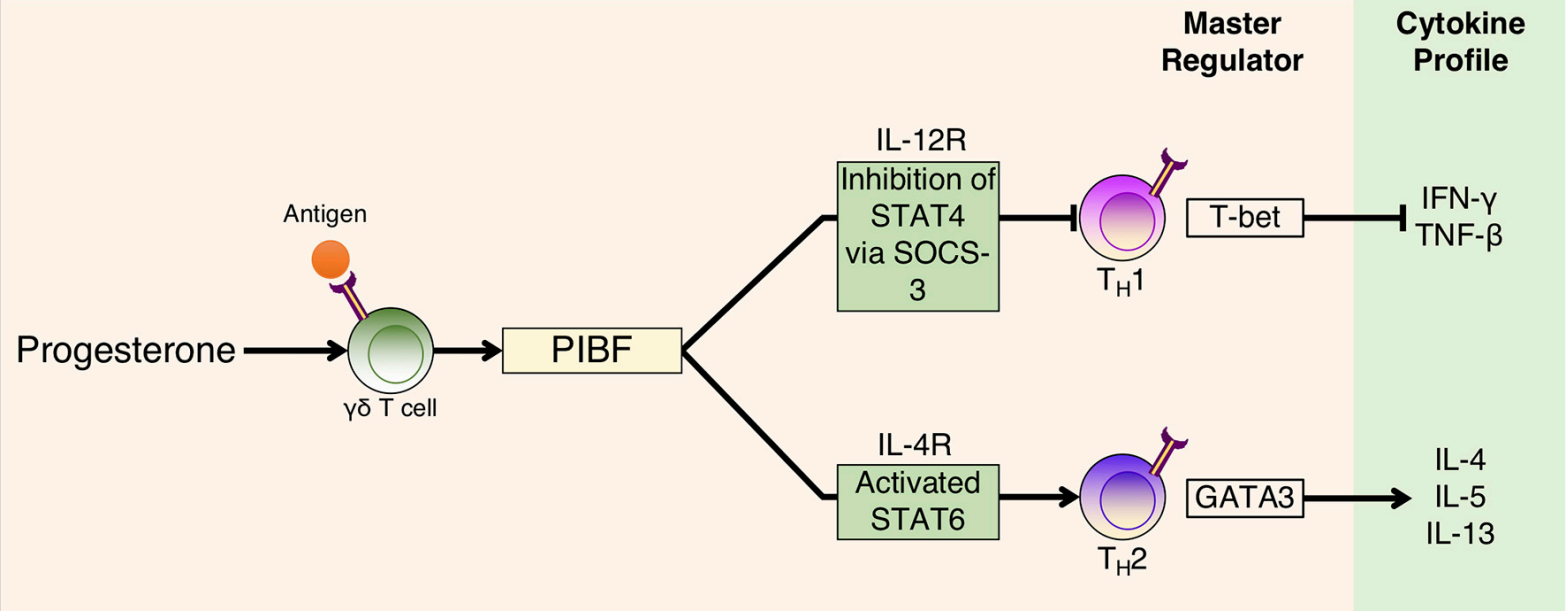
P4-dependent PIBF action on T cell differentiation and cytokine production. P4 binds to PR found in P4-sensitive γδ T cells that are activated having already interacted with fetal/paternal antigen. PIBF is subsequently released, which binds to the PIBF receptor, which is heterodimerized with the IL-4 receptor, to activate the STAT6 transcription pathway for Th2 cytokine production. On Th1 cells, PIBF acts to inhibit the STAT4 pathway by promoting SOCS-3 interaction with the IL-12R receptor to inhibit Th1 cytokine production. Adapted with permission from Shah (278).

Th2 cytokines are induced by PIBF binding to its receptor whilst heterodimerized with IL-4R to result in phosphorylation of Jak1, which activates signal transducer and activator of transcription (STAT) 6 and suppressor of cytokine signaling 3 (SOCS-3) protein. SOCS-3 binds to IL-12R to inhibit STAT4 phosphorylation (Figure 2) (261, 279), which is an effect that is abrogated with the use of an anti-IL-4R antibody (261). A consequence of this Th2 cytokine bias is the modulation of humoral immune responses. For example, PIBF exposure to *in vitro* cultures of hybridoma cells can cause the expression of PIBF in lymphocytes to be associated with an increase in asymmetric antibody production, which has the potential to provide fetal protection during pregnancy (249, 280).

The effects of P4 are not limited to Th2 cytokine production. Originally, Szekeres-Bartho et al. also showed that both P4 and PIBF prevent PGF2α synthesis, and PIBF appears to do this by inhibiting phospholipase A2 activity, which leads to reduced arachidonic acid concentrations (27). PGF2α promotes myometrial smooth muscle contractions and so PIBF-driven reduction in PGF2α stimulation of myometrial cells potentially helps to maintain uterine quiescence. However, PGs can mediate alternative mechanisms of P4-associated immunomodulation (281, 282). For example, in allergic COX-2 knock-out mice, synthetic PGF2α and PGI2 promote Th17 cell differentiation of CD4^+^ T cells *in vitro* (283). Similarly, at sites of inflammation in mice where PGE2 is abundant and T cell responses are Th17-skewed, PGE2 serves to shift the cellular production of IL-17A to IFN-γ in order to promote Th1 cell proliferation and cytokine production (284). Therefore, in contrast to PGF2α, PGE2 inhibits effector T cell function and proliferation whilst promoting Treg differentiation, thus initiating and resolving mucosal inflammation (281, 284). In fact, in murine studies, arachidonic acid has been shown to upregulate activated PGE2-producing DCs that are associated with increased FoxP3^+^ Tregs and reduced T cell proliferation (285).

### Anti-cytolytic Activity

In addition to promoting a Th2 dominant cytokine profile, P4 also can upregulate HLA-G expression on trophoblast cells that mediate γδ T cell activation and assist the invading trophoblast tissue to evade host defenses, such as decidual NK (dNK) cells, by acting as a ligand for inhibitory receptors expressed on these cells (10, 269). Human uNKs express GR and not PR, and P4 appears to suppress NK cell activation and IFN-γ production *via* GR activity by altering STAT4 and IκB phosphorylation (286). PIBF-expressing granulated cells have been observed in mouse decidua and PIBF colocalizes within the cytoplasmic granules of dNK cells (234). PIBF may also have a direct action on NK cell efficacy in pregnancy; *in vitro* experiments on decidual mononuclear cells have shown that both P4 and PIBF decrease the cytotoxic activity of decidual lymphocytes and block their perforin release in a concentration-dependent manner (9). Furthermore, *in vitro* experiments have shown that RU486 decreases PIBF mRNA expression in a human leukemia cell line and this anti-progestin can improve survival and quality of life when used *in vivo* on mice with lymphocytic leukemia (183, 287). However, our own studies with women given RU486 treatment during early pregnancy have demonstrated that P4 may broadly suppress antigen-specific memory cytotoxic T lymphocyte (CTL) responses in peripheral blood (160). At the same time, contrary to previous reports, our flow cytometric data from these patients indicates this effect is not driven by PIBF (9, 272). It is possible that there was a lack of PIBF sensitivity in our patient cohort. It is also conceivable that PIBF sensitivity may simply be a property of decidual leukocytes in humans because P4 concentrations are higher in the decidua than peripheral blood (288), by virtue of their proximity to the placenta, which could result in different P4 response profiles during pregnancy.

## Concluding Remarks

**Figure 3:**
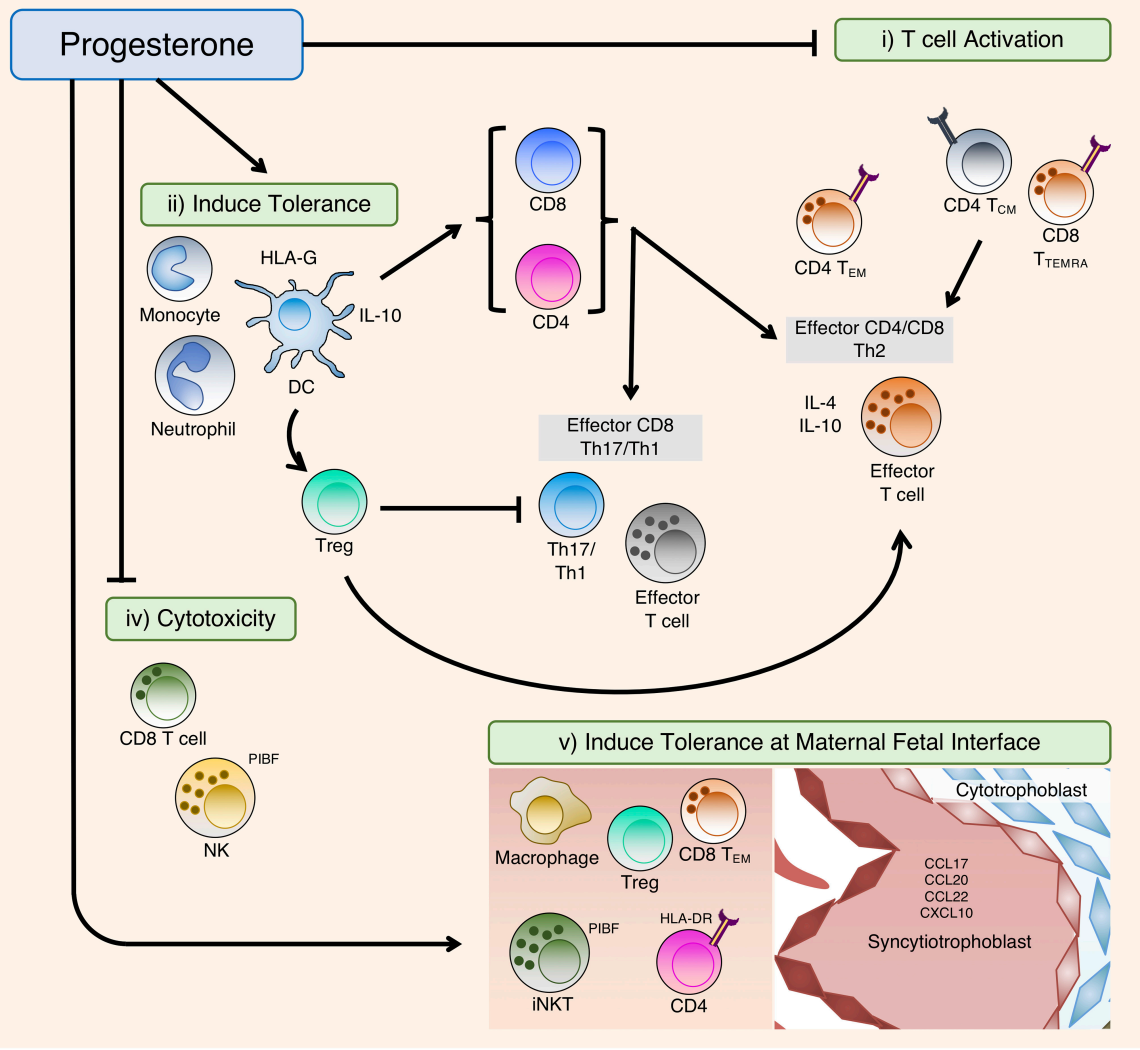
Summary of the immunomodulatory effects of P4. In the periphery, P4 affects T cell activation and differentiation directly by (i) T cell activation: modulation of T cell receptor (TCR) signal transduction or (ii) Induce tolerance: inducing tolerant antigen-presenting cells (APCs) including DC that suppress T cell activation during TCR engagement, as well as indirectly *via* induction of Tregs that subsequently modulate T cell differentiation. (iv) Cytotoxicity: P4 can also suppress cellular cytotoxicity, predominately *via* PIBF to block degranulation. (v) Induce tolerance at maternal fetal interface: At the maternal fetal interface, P4 can promote placental tissue growth and invasion by inducing immune-tolerant phenotypes of macrophages, natural killer (NK) and T regulatory (Treg) cells, whilst promoting exhaustion of activated CD4 and CD8 T cells that have interacted with placental-derived fetal-paternal antigens. Migration of these tolerant leukocytes to the maternal fetal-interface are partly driven by chemoattractant molecules expressed on placental tissue.

P4 mediates a series of immune adaptations that preferentially promote continued pregnancy (Figure 3). It achieves this by inducing a Th2 dominant cytokine environment whilst concurrently suppressing pro-inflammatory immune responses. This occurs both systemically and locally at the maternal-fetal interface. Balanced interplay between the neuroendocrine and immune compartments is a key to establishing and regulating these crucial immunomodulatory changes during a healthy pregnancy. Functional P4 withdrawal occurs as a consequence of a predetermined gestational length, which can be altered by immune cell activity from the mother, the neonate or the maternal-fetal interface to drive the onset of labor. For most of pregnancy, P4 has to both suppress maternal responses to fetal antigens and prevent overt inflammatory responses to harmful environmental stimuli encountered by the mother. Disruption to P4 signaling that promotes the progression of pregnancy can lead to the mistiming of childbirth, which often bears adverse consequences for the health of both the mother and fetus. The design of novel effective therapies for obstetric complications will be dependent on future research that further elucidates the mechanistic details of the P4 signaling network, specifically with regards to interactions between the immune and reproductive systems that occur during both healthy and pathological pregnancies.

## Author Contributions

All authors listed have made a substantial, direct and intellectual contribution to the work, and approved it for publication.

### Conflict of Interest Statement

The authors declare that the research was conducted in the absence of any commercial or financial relationships that could be construed as a potential conflict of interest.
